# Comorbidity borderline-narcissistic personality disorder

**DOI:** 10.1192/j.eurpsy.2023.790

**Published:** 2023-07-19

**Authors:** K. Douk, I. Belabess, I. Hanine

**Affiliations:** 1 psychiatry, Military hospital Mohammed V, Rabat; 2 psychiaty, psychiatric hospital Ar-Razi, salé, Morocco

## Abstract

**Introduction:**

borderline personality disorder, which is characterized by major impulsivity and marked instability of emotions, interpersonal relationships and self-image, may be associated with traits such as grandiosity, sense of entitlement, exploitation of others, over-reliance on the admiration of others to regulate self-esteem arrogant and haughty behaviors, exhibitionism and lack of empathy, traits belonging to the narcissistic personality disorder, thus modifying the clinical expression, influencing the occurrence of possible additional comorbidities and complicating both the diagnostic approach, the therapeutic strategy, the possible complications and the prognosis.

**Objectives:**

to shed light on this combination, its characteristics, its manifestations and clinical implications and its therapeutic approaches

**Methods:**

We have performed a systematic review of litterature using the following keywords on the GoogleScholar and PUBMED database: borderline personality disorder subtypes, borderline personality disorder comorbidities; borderline and narcissic comorbidity; borderline treatment

**Results:**

The authors have noted that patients meeting both borderline and narcissistic personality disorder criteria are significantly less likely to be hospitalized in psychiatric facilities and have fewer psychiatric comorbidities than those with borderline personality disorder alone, particularly anxiety disorders.

In contrast to the rarity of psychiatric complications, the authors noted that patients with both borderline and narcissistic personality criteria were more likely to exhibit other pathological personality traits, including schizotypal, histrionic and paranoid.

The authors have noted that patients meeting both borderline and narcissistic personality disorder criteria are significantly less likely to be hospitalized in psychiatric facilities and have fewer psychiatric comorbidities than those with borderline personality disorder alone, particularly anxiety disorders.In contrast to the rarity of psychiatric complications, the authors noted that patients with both borderline and narcissistic personality criteria were more likely to exhibit other pathological personality traits, including schizotypal, histrionic and paranoid.

**Conclusions:**

The combination of narcissistic personality traits and borderline personality disorder has a significant impact on clinical manifestations, complications and prognosis, which is seen positively with a decrease in psychiatric complications, self-harm and suicide attempts, hence the rarity of hospitalization compared to subjects with borderline personality disorder, thus classifying narcissistic personality traits as a protective factor for subjects with borderline personality disorder.

**Disclosure of Interest:**

None Declared

